# Domestic Correspondence

**Published:** 1884-03

**Authors:** 


### Domestic Correspondence.

### ARTICLE XIX.

MIFFLINTOWN, PA., JAN. 17, 1884.

MESSRS. EDITORS :—I was called, the night before last, to see a man and his wife who had been poisoned by eating Chicago canned corn beef. The circumstances were these :

The man is a laborer on the railroad. He returned from his work at 7 o'clock P. M. Shortly afterward they ate their supper, of potatoes, bread, and canned corned beef. At about 10 o'clock P. M., the wife was taken with sick stomach, vomiting, pain in stomach, dryness of throat, thirst, shortness of breath, weak and rapid pulse, and finally purging.

Between 11 and 12 o'clock, the man was taken with the same symptoms. I was sent for. Between 1 and 2 o'clock A. M. the violent symptoms subsided, and they made a speedy recovery.

The man stated that when he opened the can, he noticed steam or vapor escape from it. He said the meat tasted all right. I am unable to say whether the poisoning was from the beef, or from tin or lead. The symptoms were certainly those of tin or lead, but it is possible that imperfectly canned beef might undergo such change as to produce similar effects.

A few years ago, some persons here were poisoned in the same manner. THOMAS A. ELDER.

---

THE NORTHWESTERN MEDICAL COLLEGE.

EDITORS CHICAGO MEDICAL JOURNAL AND EXAMINER :

[Special Dispatch to the St. Joseph Daily *Herald.*]

JEFFERSON CITY, MO., January 8.—The State Board of Health of Missouri, in session here to-day, fully exonerates the Northwestern Medical

College, of St. Joseph, from all charges against it, and recognizes it as standing on a level with the best schools of the State. There was a full meeting of the Board, assisted by Drs. Rauch and Taliaferro, of the Illinois Board.

Owing to the widespread publication in the secular press and the medical journals, last September, of a report that the Missouri State Board of Health had refused to recognize the diplomas of the Northwestern Medical College, thereby doing said college a grave injury and an unwarranted injustice, I transmit to you the above dispatch noting the action of the Board at its annual meeting, which, together with this note, I hope you will do me the favor to publish in your next number. It is sufficient to state further, that the information against said school, and upon which the September publications were predicated, proved, upon investigation, to be the work of an actively interested enemy of the school near at home, and one upon whose veracity any one knowing him would place no reliance.

Respectfully,

J. P. CHESNEY, M.D.,

*Secretary of the Faculty.*

St. Joseph, Mo., Jan. 15, 1884.

---

ELGIN, January, 18, 1884.

TO THE CHICAGO MEDICAL JOURNAL AND EXAMINER:

Vaccination as an immunity against small-pox I have great faith in. I also, from my own experience and observation, believe that once thoroughly done, a typical pustule is sufficient. Of course a small percentage will have varioloid after exposure. Small-pox itself will occur the second time in rare instances. My first experience with this disease as a physician was at Lake Zurich in this State. I had eleven cases, nine children, who never had been vaccinated, and the mother who had been in childhood. The mother had only varioloid. The father, who in youth had also been vaccinated escaped altogether. Two of the children died with confluent small-pox.

My next experience with this sickness was in New Orleans in the fall, winter, and spring of 1863-4. Here we had as many as

1,000 patients in hospital at a time—averaged that many for months. We were daily receiving new recruits at our wards and daily discharging them, not a few went to the grave.

Here then during all these months of hospital practice, I was in constant attendence on small-pox patients, still I was in uniformly good health and so was my family, notwithstanding I went home to them directly from the hospital daily. My wife and three daughters had been only vaccinated in youth. I am a firm believer in the efficacy of vaccination. But how many there are who think they have been vaccinated who have not been? Spurious matter has been used; or if good virus, it has been inefficiently done. The impression with many is that if they only have had a bad sore on their arm "it has took well," when in fact, in all such cases it never "took" at all. But who will pretend to portray the ill consequences of such spurious vaccination. Even when the matter is good, too much scarifying the arm lets the virus run out with the blood, making an ugly sore or ulcer, no sign of a typical pustule. This is no vaccination, even if the matter was good—a false security. I have had my attention called to the appearance of the arms in one week after the so-called vaccination, finding ouly an ugly looking sore, no signs whatever of any appearing pustule, yet they thought it was taking well because the arm was swollen and painful, as far up as into the axillary space. It has been my rule when I see a case of previous vaccination, showing a typical scar, to let what I considered well enough alone. Is is true, I suppose, that whereever vaccination shows a healthy process such might seem a risk of varioloid in case of exposure to small-pox. In these cases a paucity of matter may have been at first introduced into the system.

The immortal Jenner believed that once introducing the kine pox virus into the system properly by vaccination was sufficient. Heim asserts that there are no less than five spurious cow-pox viruses. Should not this caution us against the too frequent use of this matter? What reason have we to suppose that vaccination runs out "every seven years" or oftener. For now I believe it is getting fashionable to vaccinate every year in case there is any small-pox in the country. Why does not contagious disease seize upon us (if we expose ourselves) as often as every seven years, or every year, as the case may be? Is it not fearful to be introducing into the human system by inoculation every few months some sort of matter, of the character of which we know little or nothing? How do we know but that we are introducing seeds into the system that will poison the blood of the patients and sooner or later cause the death of them? Especially when we consider that there was not one chance out of a million of the patients ever taking the small-pox. H. ROSECRANS, M.D.

---

MR. EDITOR:

The enclosed inquiries are addressed to you by the American Association for the Cure of Inebriates, which was organized in 1870, for the purpose of studying the nature and causes of inebriety from a purely scientific standpoint.

The purpose of these questions is to gather the practical experience and observation of leading members of the medical profession, as a basis for a more accurate knowledge of the nature and character of inebriety.

Full answers to these inquiries, and other facts relating to this subject, are most earnestly solicited, and will be fully credited to each reporter. The results of this investigation will be sent to each one free when published.

Trusting to hear from you soon, I am, with thanks,

Very truly yours,

T. D. CROTHERS, M.D.,

Sec'y of Committee and Editor of *Journal of Inebriety.*

P. O. Address: Hartford Conn.

Can you give any facts, from observation, bearing on the heredity of inebriety, particularly as to the presence of Insanity, Epilepsy, Phthisis, Inebriety, or other neuroses in the parents or relatives of inebriates?

Give cases with histories if possible?

Can you give any history of inebriates whose drinking dated from or was influenced by head injuries, sunstroke, syphilis; or could be traced to mental shock, disease or injury of any kind; also, to over-work, nervous exhaustion, anæmia, and any specific causes which broke down or injured the system?

Have you seen any cases in which insanity or epilepsy either preceded or followed inebriety? If so, was it traced to the use of alcohol alone, or was it due in part, or in whole, to some inherited or acquired diathesis?

Have you noted any distinction between the different forms of inebriety, such as irregular, continuous, or periodical inebriety? State any facts you have noticed which relate to the periods and forms of drinking?

What particular mental and physical changes have you noticed concerning the character and general health of the inebriate that would suggest the idea of disease and the need of physical care and treatment?

Have you noticed any form or condition of inebriety that seems to be produced or is largely influenced by the kind of alcoholic drink used, or the work engaged in, or the food or climate, or any other unsanitary surroundings?

Illustrative cases concerning any of these inquiries will be welcome, and a full expression of opinion from observation and experience is urgently requested.

HARTFORD, CONN., Jan. 22, 1884.

---

ARCHIVES OF DENTISTRY.—An association of dentists has been formed for the purpose of publishing a dental journal under the above title. This association is being incorporated, and has purchased the *Missouri Dental Journal* as a starting-point upon which to establish the new enterprise.

The editorial work of the journal will be directed by the association, and the editorial staff will comprise representative men chosen from all sections of the country. Its course will be outspoken, fearless, and progressive, and, as its name indicates, will aim to record all important events connected with the current history of dentistry. Its pages will be open to a full and free discussion of all subjects relating to dental science and art, and also those of related sciences. An earnest effort will be made to produce a journal that will be acceptable, if not indispensable, to every dental practitioner.

The plan adopted contemplates the publication of this journal simultaneously in the cities of Chicago, St. Louis and Atlanta.

For the present, all communications should be sent to the Dental Journal Association, 48 S. Clark Street, Chicago, Ill., 405 N. Third Street, St. Louis, Mo., or 3 White Hall Street, Atlanta, Ga.

The subscription price of the new journal will be two dollars for the year 1884, and three dollars for each year thereafter.

This advance circular is sent to you with a view of informing you of the action already taken, and of soliciting your coöperation in the work.

THE DENTAL JOURNAL ASSOCIATION.

